# Nutrient-Mediated Architectural Plasticity of a Predatory Trap

**DOI:** 10.1371/journal.pone.0054558

**Published:** 2013-01-22

**Authors:** Sean J. Blamires, I-Min Tso

**Affiliations:** 1 Department of Life Science, Tunghai University, Taichung, Taiwan; 2 Center for Tropical Ecology and Biodiversity, Tunghai University, Taichung, Taiwan; USDA-Agricultural Research Service, United States of America

## Abstract

**Background:**

Nutrients such as protein may be actively sought by foraging animals. Many predators exhibit foraging plasticity, but how their foraging strategies are affected when faced with nutrient deprivation is largely unknown. In spiders, the assimilation of protein into silk may be in conflict with somatic processes so we predicted web building to be affected under protein depletion.

**Methodology/Principal Findings:**

To assess the influence of protein intake on foraging plasticity we fed the orb-web spiders *Argiope aemula* and *Cyclosa mulmeinensis* high, low or no protein solutions over 10 days and allowed them to build webs. We compared post-feeding web architectural components and major ampullate (MA) silk amino acid compositions. We found that the number of radii in webs increased in both species when fed high protein solutions. Mesh size increased in *A. aemula* when fed a high protein solution. MA silk proline and alanine compositions varied in each species with contrasting variations in alanine between the two species. Glycine compositions only varied in *C. mulmeinensis* silk. No spiders significantly lost or gained mass on any feeding treatment, so they did not sacrifice somatic maintenance for amino acid investment in silk.

**Conclusions/Significance:**

Our results show that the amount of protein taken in significantly affects the foraging decisions of trap-building predators, such as orb web spiders. Nevertheless, the subtle differences found between species in the association between protein intake, the amino acids invested in silk and web architectural plasticity show that the influence of protein deprivation on specific foraging strategies differs among different spiders.

## Introduction

Foraging models predict that animals should maximize their energetic gain from the environment while minimizing energetic output [1]. Such models may be extrapolated to incorporate macro- and micro-nutrients as currencies [2], [3]. Dietary protein has been implicated as essential for animals to regulate [2]–[5]. Indeed, experiments based on a nutritional geometric framework model have shown that herbivorous and some carnivorous animals may selectively forage to balance their uptake of protein and other nutrients when the protein content of their prey becomes perceptibly low [2], [5]–[9].

Since the type and availability of prey fluctuates spatially and temporally, predators with plastic foraging strategies may have selective advantages over predators with fixed foraging strategies [1], [10], [11]. Foraging plasticity has been documented in trap building predators, such as ant lions and web-building spiders, since their trap is the manifestation of their foraging strategy and can be easily measured [12]. While trap plasticity has been well studied in spiders in relation to variations in prey type and quantity [11], [13]–[16], how nutrient deprivation influences foraging plasticity in trap building predators is not well understood because it is difficult to decouple the influence of nutrients from the multitude of other prey attributes, e.g. size, sensory modalities, that may act as cues to induce plasticity [11], [12].

There is an association between the amount of dietary protein consumed by a web-building spider and plasticity in the architectural components of its web [11], [15], [16] and/or the physical and chemical properties of its silks [17]–[19]. Furthermore, dietary protein, or more specifically certain amino acids, is essential for growth, sustenance and reproductive output in spiders [20]–[22]. Some silk amino acids are costly, or impossible, for spiders to synthesize [17] so spiders may partition digested protein between somatic processes and silk. Thus, enforcing potential trade-offs when protein intake is limited. Protein allocation trade-offs may partly explain why spiders on diets of low or no protein significantly alter the amino acid compositions of their silks [19]. It may, accordingly, be expected that protein availability has an integral influence on the foraging strategies of web building spiders [5].

Architectural plasticity in orb webs has traditionally been determined as the mean amount of variation in architectural components such as web capture surface area, the width of the spaces between the sticky spirals (mesh size), the number of radii that traverse the spirals and the length or pattern of any decorations (stabilimenta); the conspicuous silken or inert structures added to the centre of the web by some spiders [23]. Spider orb webs are constructed from up to seven types of silk, each of which is secreted from a different gland and contributes specifically to different components of web architecture [17], [24]. The radii are composed of silks produced by the major (MA) and minor ampullate (MiA) glands. The spiral silks on the other hand are derived from the aggregate and flagelliform glands, and silks derived from the aciniform gland are used in decorations [24], [25]. The amino acid composition, hence the metabolic cost of synthesis, differs for each of these silks. For instance, MA and MiA silks are principally comprised (∼85%) of short chain, readily synthesizable amino acids such as glycine and alanine. Aggregate, and aciniform silks, on the other hand, are composed of around 60% synthesizable amino acids, having relatively high compositions of the longer chain amino acids proline, serine and glutamine [17]. More amino acids thus are required from food for assimilation into aggregate and aciniform silk than for assimilation into MA or MiA silk. The specific silk-associated costs of producing each architectural component of an orb web hence may explain why the components are differentially expressed when prey nutrient composition varies [12], [15], [16], [26].

Here we performed experiments to ascertain whether a graded reduction in protein intake influences the architectural plasticity of spider orb webs and, if so, how the intake of different concentrations of protein affects the expression of each architectural component. We fed two orb web spiders, *Argiope aemula* and *Cyclosa mulmeinensis*, one of three solutions; high, low or no protein concentration, and measured and compared their web architectural components pre- and post-feeding. Furthermore, we determined the amino acid composition of each spider’s MA silks and their mass pre- and post- feeding to ascertain whether the spiders traded-off the assimilation of amino acids into silk with assimilation into growth. We expected that MA silk amino acid compositions will vary concomitantly with the amount of protein that a spider consumes (as has been shown previously [18], [19]). As the metabolic costs of synthesizing the silks varies with their amino acid composition [17], [19] and the different architectural components rely on the synthesis of different silks [24], we expected co-variation between web architecture, MA silk amino acid composition and spider mass to signify a trade-off between protein allocation for somatic processes and silk production.

## Methods

### Ethics Statement

Ethic clearance was not required to perform this research. Capture permits were not required under Taiwan law as all collections were made outside of protected areas. We confirm that the collection locations were not privately owned and we did not collect any endangered or protected species.

### Spiders Studied and their Collection

We used two orb web spiders for our experiments, both of which are known to exhibit web and silk plasticity [27], [28]; *Argiope aemula* and *Cyclosa mulmeinensis*. *Argiope aemula* is a large (adult body length >17 mm; [29]) orb web spider that inhabits open grasslands of southeast Asia (Japan to Indonesia). It builds a two dimensional web that spans up to 500 mm in diameter on which it adds a cruciform (x-shaped) decoration composed of aciniform silk as an adult [29]. *Cyclosa mulmeinensis* is comparatively small (adult body length <6 mm) and inhabits windy, exposed shorelines and riverbanks [27] in southeast Asia. It builds a two-dimensional orb web (diameter <200 mm), which it decorates with a line of eggsacs that are hung vertically between the top of the web and the hub as an adult [30]–[32]. As a sub-adult it may add a line of detritus or silk as a decoration to its web [30].

We collected 45 adult female *A. aemula* from Wushihkeng, Taichung County, Taiwan, and 45 adult female *C. mulmeinensis* from Huwei, Yunlin County, Taiwan, between April 2010 and February 2011. We measured the body length (using calipers) and weighed (using a digital balance) all individuals upon collection in the field to make sure that similar sized spiders were used in the experiments. Spiders were returned to the laboratory at Tunghai University, Taichung, within 24 h of capture and acclimated at 25°C and 35% R.H. under a 12∶12 h light-dark cycle in 500 m*l* plastic cups with perforated mesh (diameter = 95 mm) lids for five days, during which they were fed one mealworm (*A. aemula*) or fruit fly (*C. mulmienensis*) per day. After five days acclimation all spiders were placed in 500×500×300 mm enclosures within a greenhouse receiving natural light until they built an orb web, which was subsequently measured (see ‘Web architectural measurements’).

### Protein Manipulation

Spiders were removed from their webs and we randomly assigned 15 individuals of each species to be fed one of three solutions: (i) high protein (HP), (ii) low protein (LP) or (iii) no protein (NP), over 10 days. We compared, for each species, the masses of the spiders that were assigned to each feeding regime prior to initiating feeding and found no differences (*A. aemula*: Kruskall-Wallis statistic = 5.46; *P* = 0.07; *C. mulmeinensis*: Kruskall-Wallis statistic = 1.26; *P* = 0.53). We fed the spiders solutions that varied in only protein concentration so other nutritional attributes that may induce plasticity were excluded from the experiment. We kept the spiders in their plastic cups for the whole 10 days of the experiment to prevent them from building webs and circumvent any confounding influences that previous web architectures might have on subsequent webs.

The HP solution was a mixture of 20 g of a pre-mixed chicken albumen solution [19] with 4 g of sucrose in 20 m*l* of water. The LP solution was a mixture of 10 g of the albumin solution with 6 g of sucrose in 30 m*l* of water. The NP solution was 8 g of sucrose in 40 m*l* of water. The ratios of the solutions were determined on the basis that the albumin solution contained approximately 20% protein. Accordingly, each of the solutions was, upon accounting for the amount of water in the albumin solution, a mixture of 8 g of nutrient in 40 m*l* of water. As protein and carbohydrates contain approximately similar stored energy densities (∼4 kJg^−1^) [33], the total energy across treatments was approximately similar (∼32 kJ), thereby excluding the possibility that differences in energy intake influenced web architecture in any of the treatments. The concentration of protein in the HP, LP and NP solutions was determined by the Department of Food Sciences, Tunghai University, from which we calculated their percent protein and carbohydrate. The HP solutions had, by dry weight, 55.5% protein and 28.7% carbohydrate (approximately 2∶1 protein: carbohydrate ratio) and the LP solution had 24.6% protein: 59.5% carbohydrate (approximately 1∶2 protein: carbohydrate ratio).

To feed the spiders we soaked 75 mm long cotton swabs in 1 m*l* of solution for approximately 5 min. We weighed each swab before and after soaking to ensure ∼0.1 g of food was absorbed. The soaked swabs were inserted into a fine (∼1 mm) slit cut by a Stanley knife into the centre of each cup’s mesh. The swabs were pushed approximately 75% of their length into the cup to ensure they hung rigidly in the middle of the cup. The inserted swabs were removed and re-weighed after one day and replaced. We determined the amount of food consumed per unit weight for each spider, accounting for evaporation, as the change in weight of the swab post-feeding less that of a swab soaked with ∼0.1 g of the same solution and left in a cup for 1 day without being fed from by a spider. We found no significant difference between treatments in the amount of food consumed per unit spider weight in either species (*A.aemulae*: Kruskall-Wallis statistic = 2.84; *P = *0.24; *C. mulmeinensis*: Kruskall-Wallis statistic = 1.59; *P* = 0.49). After completing the feeding experiment we re-weighed all spiders and placed them back in their enclosures until they built an orb web.

### Web Architecture Measurements

We observed the spiders placed in enclosures pre- and post-feeding hourly between 0600 h and 2000 h (as neither of these species builds webs at night) and noted if a complete orb web had been built. We then estimated the time taken to build webs (hours and minutes) to account for it as a potential factor influencing the proceeding parameters. We counted and measured, using a measuring tape, the following architectural components of every web: (1) the number of radii and spiral threads along the four cardinal directions (up, down, left, right), from which we calculated mesh size using a formula [14], (2) hub and web radius along the four cardinal directions, in order to estimate the web capture area [34], and (3) the total length of decorations added to the web (no decorations being recorded as 0).

### Silk Amino Acid Compositions

To determine whether the spiders varied their web architecture or mass simultaneously with variations in silk amino acid composition we collected MA silks directly from the spinnerets of spiders both pre- and post-feeding by force-silking using a mechanical spool reeled at a constant speed (1 m min^−1^) for 1 h (see [19], [28], [35] for details of the procedure). We weighed the silk from each individual to the nearest 0.01 mg on an electronic balance before placing it into 100 µ*l* Eppendorf tubes and submerged in 99% hexoflouro-isopropanol solvent (500 µ*l* of per mg of silk). The samples were subsequently hydrolyzed in 6 mol l^−1^ HCl for 24 h and the composition of glutamine, serine, proline, glycine and alanine, i.e. the amino acids representing >90% of the total amino acids in MA silks in these genera of spiders [36], was determined by high performance reverse-phase liquid chromatography (Waters Pico-Tag Amino Acid Column, Milford CA, USA).

### Statistical Analyses

All data pertaining to web architectural parameters and web construction times had heterogeneous variances (Levene’s tests; *P*<0.05) and did not conform to normality (Kolomogrov-Smirnov tests; *P*>0.05), even upon transformation (log_10_, SQRT, or arcsine), so we used a series of Friedman’s non-parametric ANOVAs [37] to compare the: (i) number of radii, (ii) mesh sizes, (iii) capture area, (iv) decoration length, (v) construction time of webs, and (vi) spider mass across treatments for both *A. aemula* and *C. mulmeinensis* post-feeding webs. We used Tukey’s HSD *post-hoc* tests to identify the differing variables when significance among treatments was detected.

For each species, we identified any MA silk amino acids that significantly varied in composition pre- compared to post-feeding by a series of paired (within individuals) Kruskall-Wallis tests. We used a multiple regression model, incorporating all of the data across treatments for each species, to ascertain the relationships between amino acid compositions in the silks that varied in composition, the significantly varying web architectural parameters (determined as described above), web construction time and spider mass. All data were tested for normality, linearity, homoscedasticity, and singularity using Q-Q scatter plots, transforming (log_10_ or SQRT) data where necessary.

## Results

**Figure 1:**
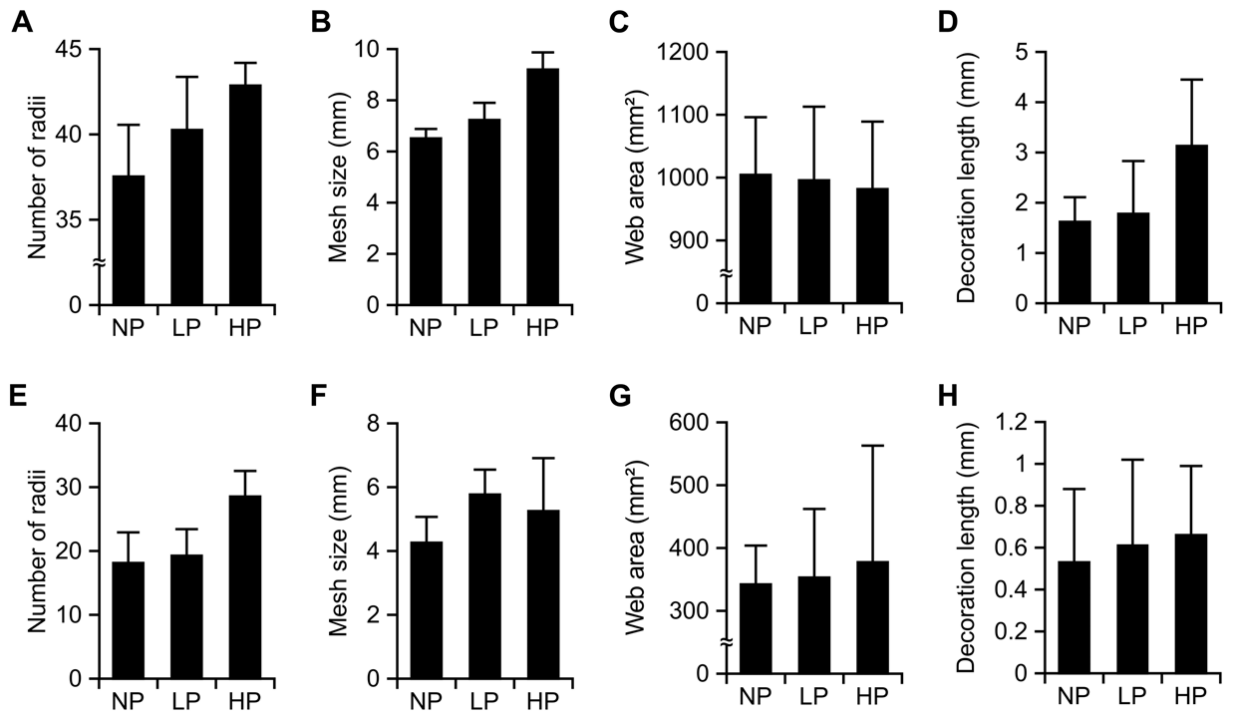
Mean (± s.e.) number of radii (A, E), mesh size (B, F), web area (C, G), and decoration length (D, H), for webs of *Argiope aemula* (A–D) and *Cyclosa mulmeinensis* (E–H) when they had been fed solutions of no protein (NP), low protein (LP) or high protein (HP) concentration.

**Figure 2:**
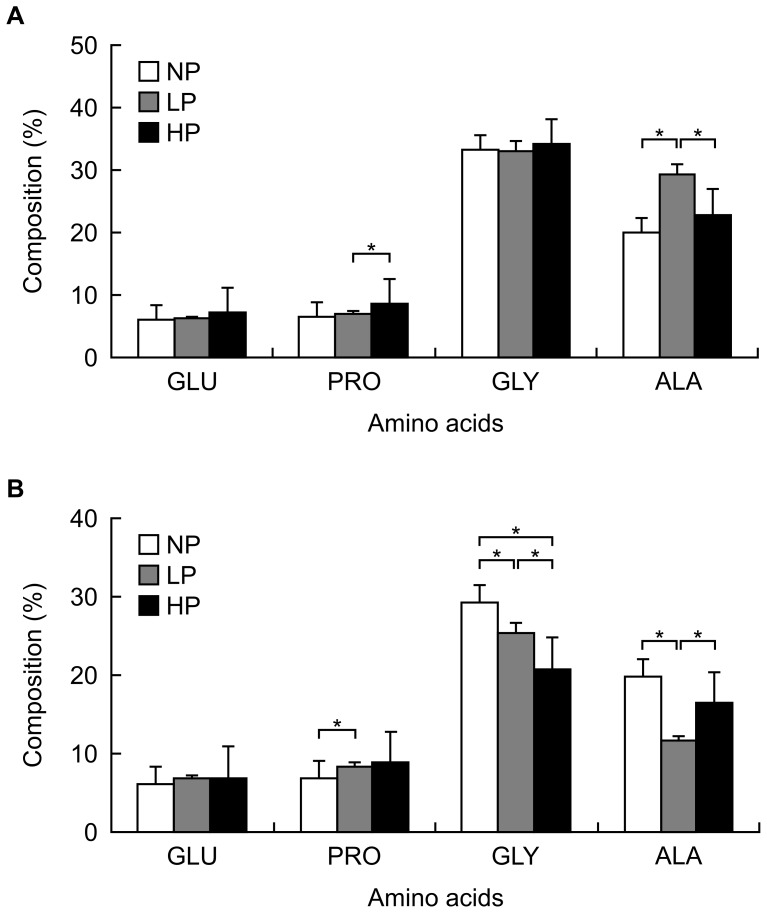
Amino acid (GLU = glutamine, PRO = proline, GLY = glycine, ALA = alanine) compositions in post-treatment MA silks, for *Argiope aemula* (A) and *Cyclosa mulmienensis* (B). * indicates significant differences (*P*<0.05) were detected by a Kruskall-Wallis tests between treatments.

In *Argiope aemula* webs the number of radii, mesh size and decoration length differed between treatments (Table 1). Spiders fed the HP treatment built webs with significantly more radii, significantly wider mesh sizes and longer decorations than those fed the LP treatment (Tukey’s HSD; *P*<0.05; Fig. 1A, B, D). Spiders fed the LP treatment had significantly more radii and significantly wider mesh sizes than those fed the NP treatment (Tukey’s HSD; *P*<0.05; Fig. 1A, B). Web capture area, time taken to build a web and spider mass were unaffected by the feeding treatments (Table 1; Fig. 1C). In *Cyclosa mulmeinensis* webs, the number of radii also differed between treatments, with spiders fed the HP treatment having significantly more radii than both the LP and NP treatments (Table 1; Tukey’s HSD; *P*<0.05; Fig. 1E). Mesh size, web capture area, decoration length, time taken to build a web and spider mass were unaffected by the feeding treatments (Table 1; Fig. 2F–H).

**Table 1:** Results of Friedman’s non-parametric ANOVAs and Tukey’s HSD *post-hoc* comparisons for (A) *Argiope aemula* and (B) *Cyclosa mulmeinensis*, comparing feeding treatment [high (HP), low (LP) or no (NP) protein] influences on (i) the number of radii (#radii), (ii) mesh size, (iii) capture area, (iv) decoration length, (v) time taken to build a web, and (vi) post-feeding spider mass.

Parameter	Friedman’s statistic	Kendall’s co-efficient of concordance	p	Post-hoc comparison
*A. aemula*				
# Radii	8.776	0.290	0.012	HP> LP> NP
Mesh size	8.533	0.284	0.014	HP> LP> NP
Web area	0.408	0.133	0.810	–
Decoration length	9.513	0.332	0.010	HP> LP = NP
Time to build	1.689	0.056	0.430	–
spider mass	4.933	0.164	0.080	–
*C. mulmeinensis*				
# Radii	7.001	0.437	0.030	HP> LP = NP
Mesh size	2.253	0.141	0.325	–
Web area	4.750	0.297	0.093	–
Decoration length	0.608	0.205	0.730	–
Time to build	1.932	0.064	0.382	–
**Spider mass**	1.752	0.109	0.417	–

The composition of MA silk proline and alanine differed according to feeding treatment for both *A. aemula* (proline: Kruskall Wallis statistic = 22.023; *P*<0.01, alanine: Kruskall Wallis statistic = 19.25; *P*<0.01; Fig. 2A; *N* = 45) and *C. mulmeinensis* (proline: Kruskall Wallis statistic = 9.099; *P* = 0.03, alanine: Kruskall Wallis statistic = 11.25; *P* = 0.01; Fig. 2B). The influence of treatment on MA silk alanine composition, however, contrasted between the two species (c.f. Fig. 2A–B). The composition of glycine also varied according to feeding treatment in *C. mulmeinensis* silk (Kruskall Wallis statistic = 23.21; *P*<0.01; Fig. 2A). The number of radii in *A. aemula* webs was positively correlated with proline composition of its MA silk (Table 2). The number of radii in *C. mulmeinensis* webs, likewise, correlated positively with MA silk proline composition and it correlated negatively with MA silk alanine composition (Table 3).

**Table 2:** Multiple regression models for *Argiope aemula* between number of radii, mesh size and proline and alanine compositions in post-feeding webs.

	#Radii				Mesh	size		
Amino acid	β	SE	t_40_	p	B	SE	t_40_	p
PRO	0.62	0.13	0.31	0.03	−0.25	0.24	0.99	0.33
ALA	0.18	0.23	0.76	0.45	0.10	0.23	0.44	0.66

**Table 3:** Multiple regression models for *Cyclosa mulmeinensis* between web architectural features and proline, alanine and glycine compositions in post-feeding webs.

	# Radii			
Amino acid	β	SE	t_40_	p
PRO	0.25	0.19	2.31	0.03
ALA	−0.52	0.17	−2.93	0.02
GLY	0.05	0.21	0.34	0.73

## Discussion

While recent studies have shown, using a geometric framework, that spiders and other predators may forage in a way that balances the intake of specific nutrients [4], [5], [9], the foraging decisions made by predators in the light of deprivation of specific nutrients has remained largely untested. Here we fed individual spiders high, low or no protein intake while holding energy intake and other variables constant in two orb web spiders, *Argiope aemula* and *Cyclosa mulmienensis* and demonstrated that protein concentration induces foraging plasticity in these trap building predators.

Previous studies have shown that orb web spiders vary the architecture of their webs when feeding on different prey [11], [12], [15], [16]. However, a multitude of prey cues may be used to induce changes in web architecture, e.g. prey size, energy, nutrients, handling characteristics [11], [12], [15]. Since these variables tend to co-vary [11], [15], previous studies have been unable to decouple them experimentally. Our work significantly expands these studies by suggesting that orb web spiders alter the architecture of their webs [11], [14], [16] and silk amino acid composition [19], [38], [39] concurrently in response to variations in the concentration of protein taken up. The concentrations of the HP and LP solutions reflect the extremes of protein concentrations that might be naturally found in insects [5], [21], [26], so we expect our findings to reflect the kind of variability in web architecture that might be expected if these spiders were forced to vary their protein intake in the field.

We found that the concentration of protein consumed influenced the radii investment in the webs of both *A. aemula* and *C. mulmienensis*, with the greatest number of radii found when the spiders were fed a high protein diet; possibly explaining why previous studies have found that the number of radii invested in orb webs by spiders varies with the type or amount of food eaten [19], [40]. The assumption that radii construction (MA silk) comes at an energetic cost that is satisfied when adequate food or food of adequate energy/nutrient quality is consumed [15], [16], [19] partially explains these findings. Nonetheless, variations in radii number in spider orb webs are often correlated with variations in other architectural parameters, for example mesh size [11], [14], [15], [41]. We found that mesh size co-varied with radii number in *A. aemula* but not *C. mulmeinensis* as a response to variations in the concentration of protein consumed. It, thus, appears that the strategic reason for the alteration in radii investment with the concentration of protein taken in differed between the two species of spider.

Studies have shown that orb web spiders vary the mesh size and capture area of their webs upon exposure to a multitude of non-nutritional prey cues, including radii-propagated vibrations of specific frequencies [11], [42]. It appears from our findings that the cues used to vary specific web components differs in different spiders; for example, *A. aemula* altered mesh size in response to nutritional cues but *C. mulmeinensi* altered mesh size in response to non-nutritional cues [27]. The latter species, incidentally, inhabits exceptionally windy locations and exhibits web and silk plasticity in response to changes in wind speed [27] so it may vary its web architecture directly in response to environmental cues rather than nutritional cues as these are more imperative for its survival. We, nonetheless, note that the investment by orb web spiders in the flagelliform and aggregate silks that make up their sticky spirals is largely dependent on the silks being consumed and their compounds recycled into successive webs [17], [24], [43]. We did not enable the spiders to recycle webs in our experiments, so there may have been substandard investment in these silks by both species across all of the treatments.

A reduction in the number of radii was found for both species when feeding on the low or no protein concentration solutions. Explanations for this phenomenon might include: (1) the mechanical performance consequences for MA silk as a result of a reduction in proline composition [19], and (2) protein intake directly constraining radii investment because a reduction in the availability of certain amino acids has stressed the spider’s protein or energy reserves causing a conflict between the assimilation of proteins into silk or somatic functions [17], [19], [21], [22]. Although spiders may have the capacity to “tune” their MA silk properties post-secretion to compensate for any proline-induced performance variations [33] stiffer radii will inevitably be deposited if proline composition decreases inimitably [44]. These stiffer radii may cause the web to become unable to adequately absorb the kinetic energy of flying prey [17], rendering explanation (1) likely. Furthermore, the mass of all spiders remained relatively unchanged throughout our experiment so explanation (2) seems relatively unlikely. Whatever the proximal basis, the reduction in the number of radii and the probable attenuation in web elasticity under protein deprivation certainly affects the web’s performance [11], [17], [43]. Additionally, the capacity to propagate tactile cues to the spider from the web periphery may be compromised when the number of radii used decreases, possibly constituting a reduction in the spider’s ability to rapidly detect and locate prey within the web [11], [45].

We found, as we had expected, a shift in MA silk amino acid compositions when different protein concentrations were taken up, but the amino acids that were affected in each species differed. Proline and alanine compositions varied in *A. aemula* MA silk, while proline, alanine and glycine compositions varied in *C. mulmeinensis* MA silk. The two-spidroin (MaSp) model describes MA silk as comprising of a mixture of two proteins, MaSp1 and MaSp2 [17], [19], [46]–[50]. MaSp1 consists of alanine and glycine repetitive motifs. MaSp2 on the other hand contains additional proline-containing motifs as well as significantly more glutamine than MaSp1 [46], [47], [49]. A shift in the relative expression of these proteins (e.g. less MaSp2 to more MaSp1) may explain why *C. mulmeninensis*’ MA silk decreased in proline and glutamine composition while concurrently increasing in alanine and glycine composition when protein intake reduced. The model, however, cannot explain why *A. aemula*’s MA silk decreased in both proline and alanine composition (at least for the HP compared to NP treatments) when protein intake was reduced. Perhaps different proteins (see [50]) are expressed in the MA silk of *A. aemula* compared to *C. mulmeninensis*. Alternatively, the same proteins in *A. aemula* and *C. mulmeinensis* silk may be regulated under the influence of protein intake in different ways. More information on the silk proteins expressed by these species is required to ascertain why the plastic responses of their silks differed under similar manipulations of protein intake.

We found that decoration length varied with variations in protein intake concentration in *A. aemula* but not *C. mulmeinensis*. The finding of significant variations in decoration length in *A. aemula* is in agreement with a finding that *Argiope keyserlingi* fed protein-enhanced flies increased the length of its silk decorations compared to those fed protein-poor flies [26]. Nevertheless, the influence of energy intake or the behaviour of the flies could not be ruled out as having an influence in that study. Our finding, however, suggests that protein concentration induces the response. The results of Blamires et al. [26] and those herein suggest that aciniform silk is costly to synthesize and used less when protein intake is restricted. Aciniform silk’s high proportion of long chain amino acids, such as proline, serine and glutamine [17], [24], concur with a probable high synthesis cost. Decoration design and use in *Argiope* spp. has also been predicted to vary in response to non-nutritional cues [12], [51], [52]. Nevertheless we expect, on the basis of our finding of congruence between decoration variation and protein taken in, that nutrients are of foremost importance. The use of decorations in *C. mulmeinensis* was variable and sporadic, as it is for other *Cyclosa* spp. [30], [53], [54], and not affected by variations in protein intake. The precise cues that initiate differential expression of web decorations in this genus thus remain elusive and warrant further investigation.

In summary, we found that a reduction in protein intake induces variations in web architecture and MA silk amino acid composition in two orb web spiders, providing evidence that nutrients act as a cue to induce foraging plasticity among trap building predators. Nonetheless, we found dissimilarity in the specific architectural variations that differed with protein intake in the two spiders. Neither species sacrificed body mass at the expense of MA silk amino acid composition or investment in web components, so a trade-off between somatic maintenance and silk is not implicit. Orb web spider MA silks are predominantly composed of short chain synthesizable amino acids [17], [19], [24], hence, balancing the allocation of dietary protein between somatic maintenance and silk is probably not imperative as long as the spider continues to ingest protein. Under protein depletion a conflict in protein allocation between silk and somatic processes seems to be avoided because silk amino acid composition is altered, saving protein and energy for somatic processes. Post-secretion processing of the silk may ensure maintenance of the functionality of the silk despite a change in amino acid composition [35]. Protein is, henceforth, an important nutrient for web building spiders to regularly consume. While there is evidence that this is true for other predators [4], [5], [7], [9], more studies are required to ascertain how universally applicable it is.
